# Nephrobronchial fistula a case report and review of the literature

**DOI:** 10.1016/j.radcr.2021.08.025

**Published:** 2021-09-08

**Authors:** Stefania Tamburrini, Valeria Fiorini, Marina Lugarà, Giorgio Napodano, Dario Del Biondo, Fiorenzo Squame, Giuseppe Sarti, Pasquale Quassone, Maria Gabriella Coppola, Michele Iannuzzi, Mario Di Stasio, Olena Shatalova, Ines Marano, Lucio Cagini

**Affiliations:** a Department of Radiology, Ospedale del Mare, ASL NA1 Centro, Naples, Italy; b Department of Internal Medicine, Ospedale del Mare, ASL NA1 Centro, Naples, Italy; c Department of Urology, Ospedale del Mare, ASL NA1 Centro, Naples, Italy; d Department of Nuclear Medicine, Ospedale del Mare, ASL NA1 Centro, Naples, Italy; e Department of Radiology, "Università degli Studi della Campania Luigi Vanvitelli", Naples, Italy; f Department of Anesthesiology and Intensive Care, Ospedale del Mare, ASL NA1 Centro, Naples, Italy; g Department of Thoracic Surgery, Ospedale del Mare, ASL NA1 Centro, Naples, Italy

**Keywords:** Nephrobronchial fistula, Xanthogranulomatous pyelonephritis, Abscess, Computed tomography

## Abstract

Nephrobronchial fistula is an extremely rare complications of renal infections. We present a case of nephrobronchial fistula in a middle age immunocompetent woman who complained cough and weight loss, with underlying asymptomatic nephrolithiasis. She underwent a chest X-ray that showed left lower lobe infiltrate and abdominal ultrasound. Abdominal ultrasound showed a complicated pyonephrosis ; CT of chest-abdomen-pelvis with intravenous contrast was performed in order to stage and define the extension of the pathology. At CT, a suspected diagnosis of stage III xanthogranulomatous pyelonephritis complicated with pyonephrosis and a nephrobronchial fistula was formulated. A nephrostomy tube was placed, and the patient was treated with antibiotics. Follow up CT, performed after 15 days, showed the healing of the fistulous connection between the perinephric abscess and bronchi; the patient underwent nephrectomy with no airway complication during intubation. Histopathological diagnosis confirmed the presence on complicated xanthogranulomatous pyelonephritis.

## Introduction

Pulmonary complication occurs in up to 20% or infectious renal diseases with renal and/or perirenal abscesses [1], [2], [3], [4]. The preference for extension of inflammation superiorly through the diaphragm was firstly described by Evans in 1991 [5] who hypothesized that the lines of fusion of the renal fascial planes tend to direct the exudate within the retroperitoneal compartment. Among causes of pulmonary extension of renal pathologies, renal infections [6], [7], [8], complicated pyonephrosis [3,[9], [10], [11], [12], [13], [14], [15] and xanthogranulomatous pyelonephritis [16], [17], [18], [19], [20], [21], [22] represent the more frequent, tuberculosis has also been reported [23]. Nephropulmonary fistula are second in incidence only to nephrocolonic fistulas. The first case of reno-pulmonary fistula was reported by Bowditch in 1870 [24]. Nephrobronchial fistula was reported in 67 cases before 1949 [24], after 1949 the availability and diffusion of effective antibiotics and surgical techniques has decreased the complications of renal infections greatly [13]. Nephrobronchial fistula is a rare sequela of perinephric abscesses, usually occurring in adult and very occasionally in children [7]. We discuss a case of nephrobronchial fistula in a stage III xanthogranulomatous pyelonephritis.

## Case presentation

**Fig. 1:**
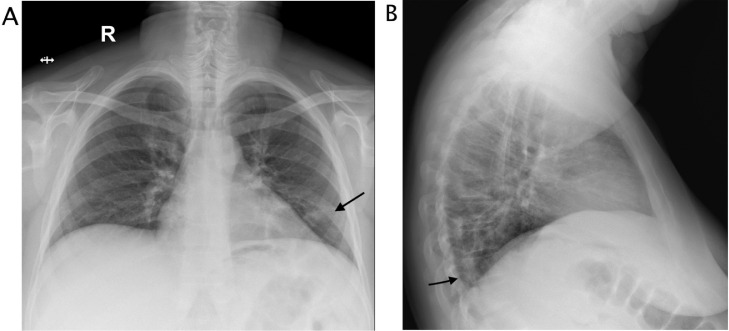
Chest X-Ray in posteroanterior (A) and lateral (B) projections. On the left basal lung, a small area of infiltrate is appreciable (black arrow). The diaphragm is well defined, no pleural effusion is detected.

**Fig. 2:**
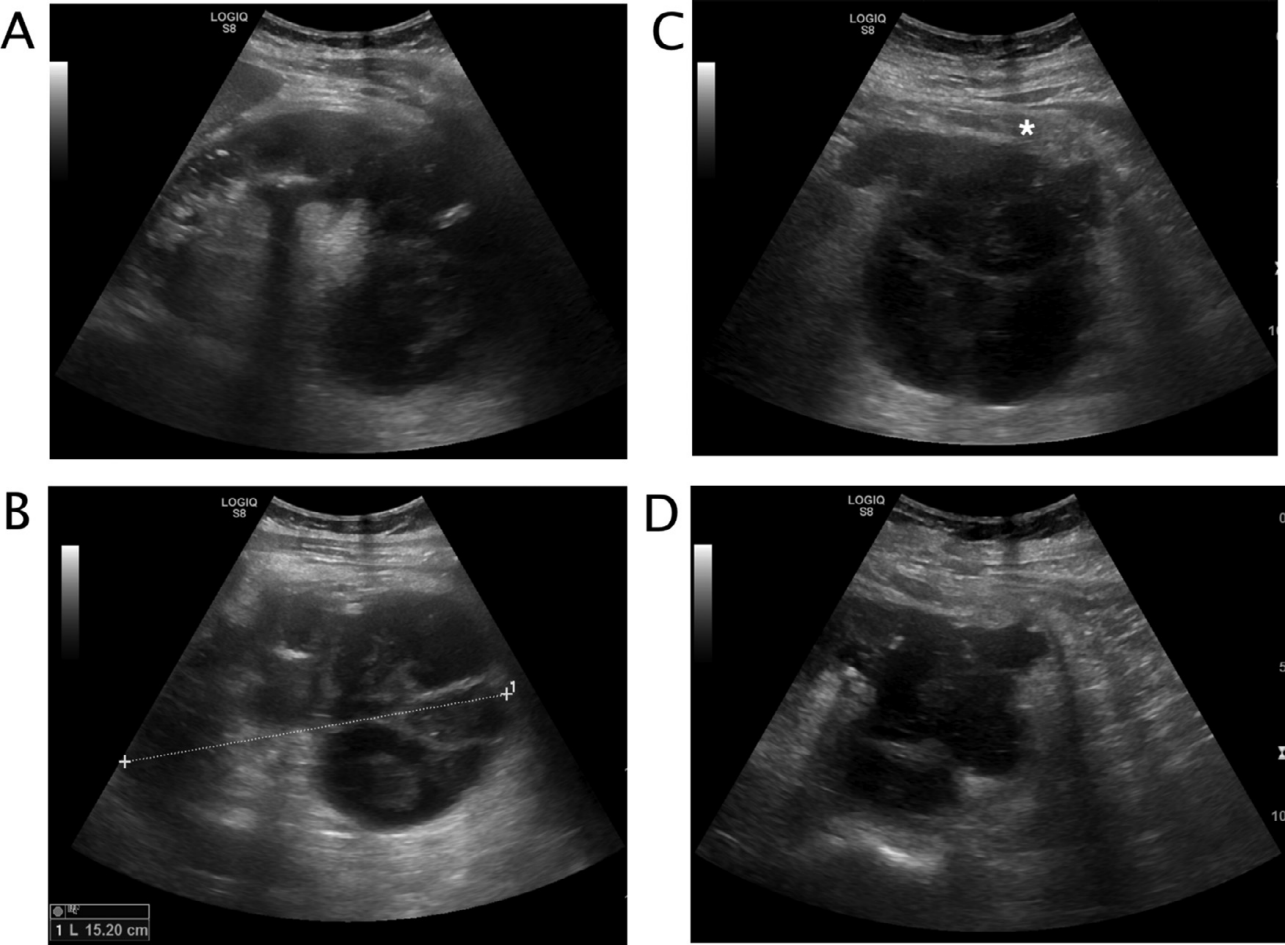
Left kidney ultrasound. The kidney is enlarged (A, B) with fluid filled dilated calico-pelvic system, within inhomogeneous hypoechogenic material is appreciable (A, C). Hyperechogenic spot are visible, referable to calculi (A, B). The outer border of the lower pole is irregular, and dilated calyceal system appear corticalized and protruding into the perirenal fat close to the perirenal fascia that appears thickened (*) (C, D). Perirenal fat is inhomogeneous and slightly hypoechogenic (D).

**Fig. 3:**
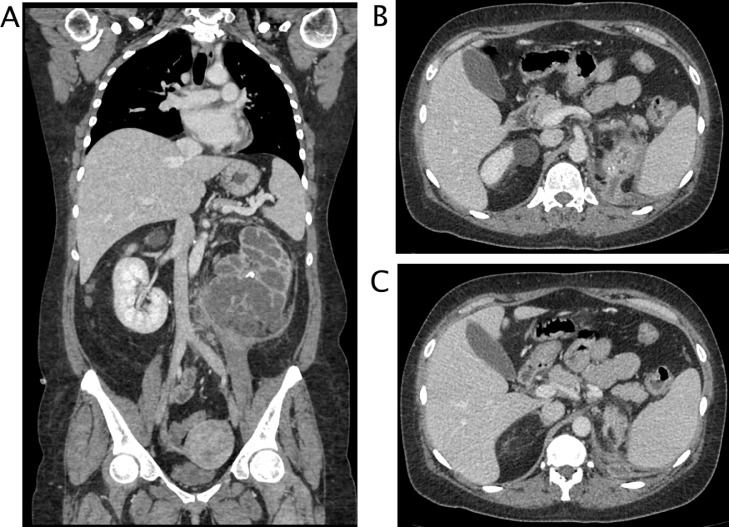
CT with intravenous contrast, parenchymal phase. (A) multiplanar coronal plane recontruction . The left kidney is enlarged, calico-pelvic system in markedly dilated with thickening of parietal wall and calyceal corticalization. Calculi are appreciable in the pelvis. Perirenal fascia is thickened and there is stranding of the perirenal fat. On the lower pole, fluid dilated calyces are not well demarcated and close to perirenal fascia. Iliopsoas muscle appears enlarged and edematous as inflamed secondarily by the infectious renal process. Nodes are detectable. (B, C) axial images. On the upper pole of the left kidney a fluid collection with hyperemic wall is appreciable, the collection goes through the diaphragm that appears focally thickened.

**Fig. 4:**
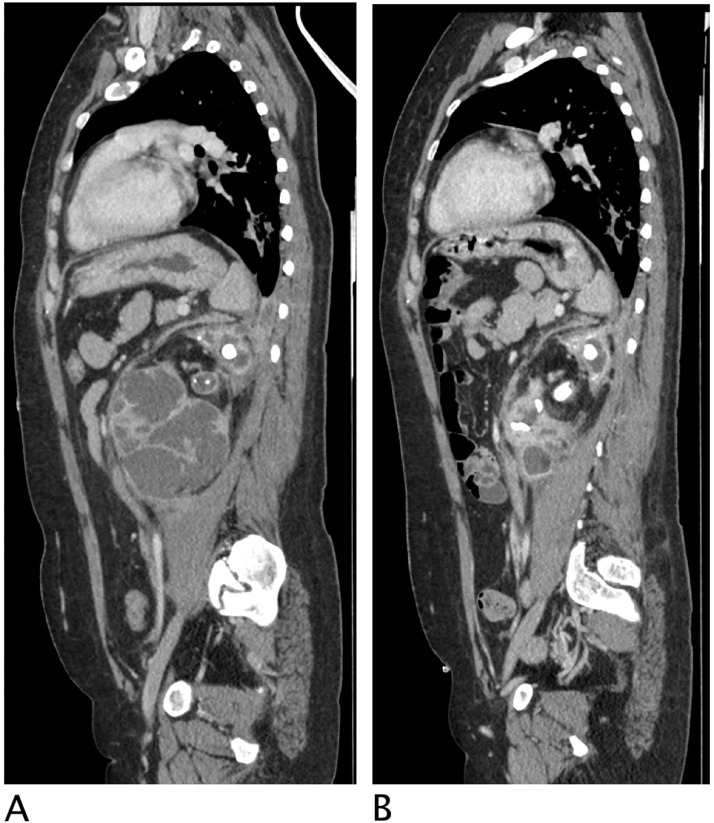
CT with intravenous contrast, parenchymal phase. Multiplanar sagittal plane reconstruction . (A) Admission CT Left markedly dilation of calico- pelvic system with calyces corticalization. , A fistulous connection is clearly visible between the upper pole of the left kidney through the diaphragm. (B) CT after 15 days of antibiotics therapy and stent placement. The fistulous connection is no more appreciable, a soft tissue band with moderate enhancement is visible. The calico pelvic system appears significantly decompressed.

**Fig. 5:**
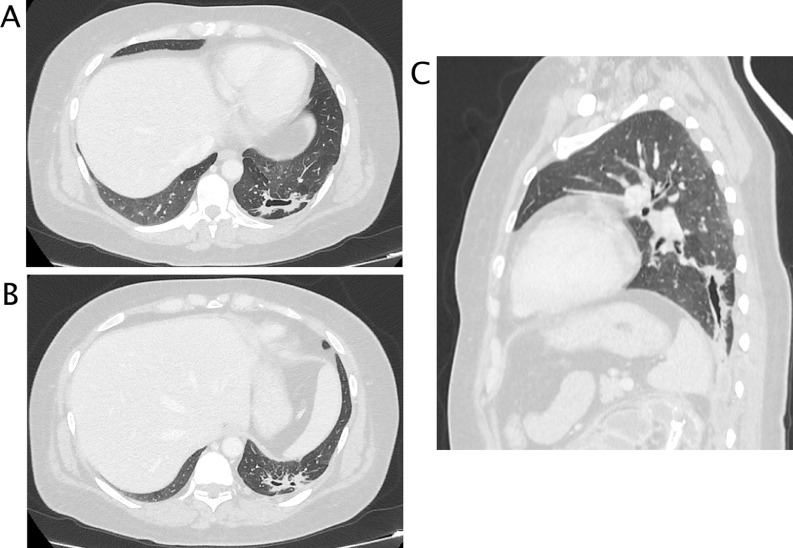
Axial (A, B) and multiplanar sagittal plane reconstruction (C) with lung window. On axial plane an air-filled cavity with irregular walls is detected on the left lower lung lobe, on sagittal (C) image the air filled lesion appears to be a focally dilated bronchi with thickened and irregular wall in direct communication through the fistulous connection with the upper left perinephric abscess.

**Fig. 6:**
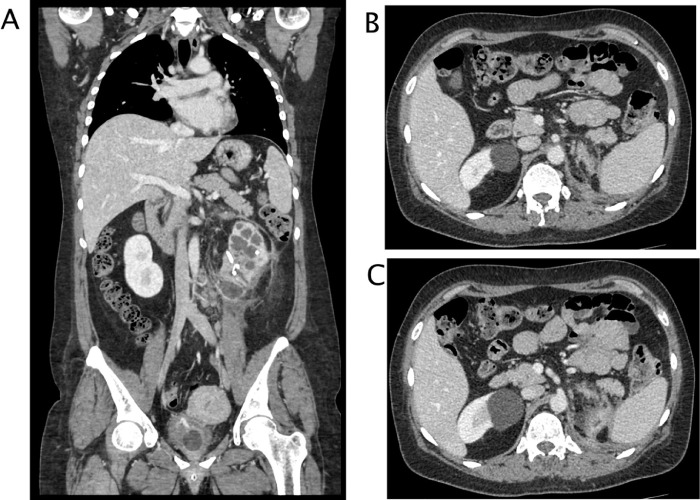
CT with intravenous contrast, parenchymal phase. Follow up CT after left nephrostomy and 15 days of antibiotic therapy. (A) multiplanar coronal plane reconstruction,.. The left calico-pelvic system is significantly decompressed, nephrostomy tube is appreciable in the pelvis. Perirenal fascia and fat are still thickened (B,C) axial images. On the upper pole of the left kidney the fluid component of the perinephric abscess is almost completely reabsorbed, a soft tissue with moderate enhancement is visible over the previously reported fistulous connection.

**Fig. 7:**
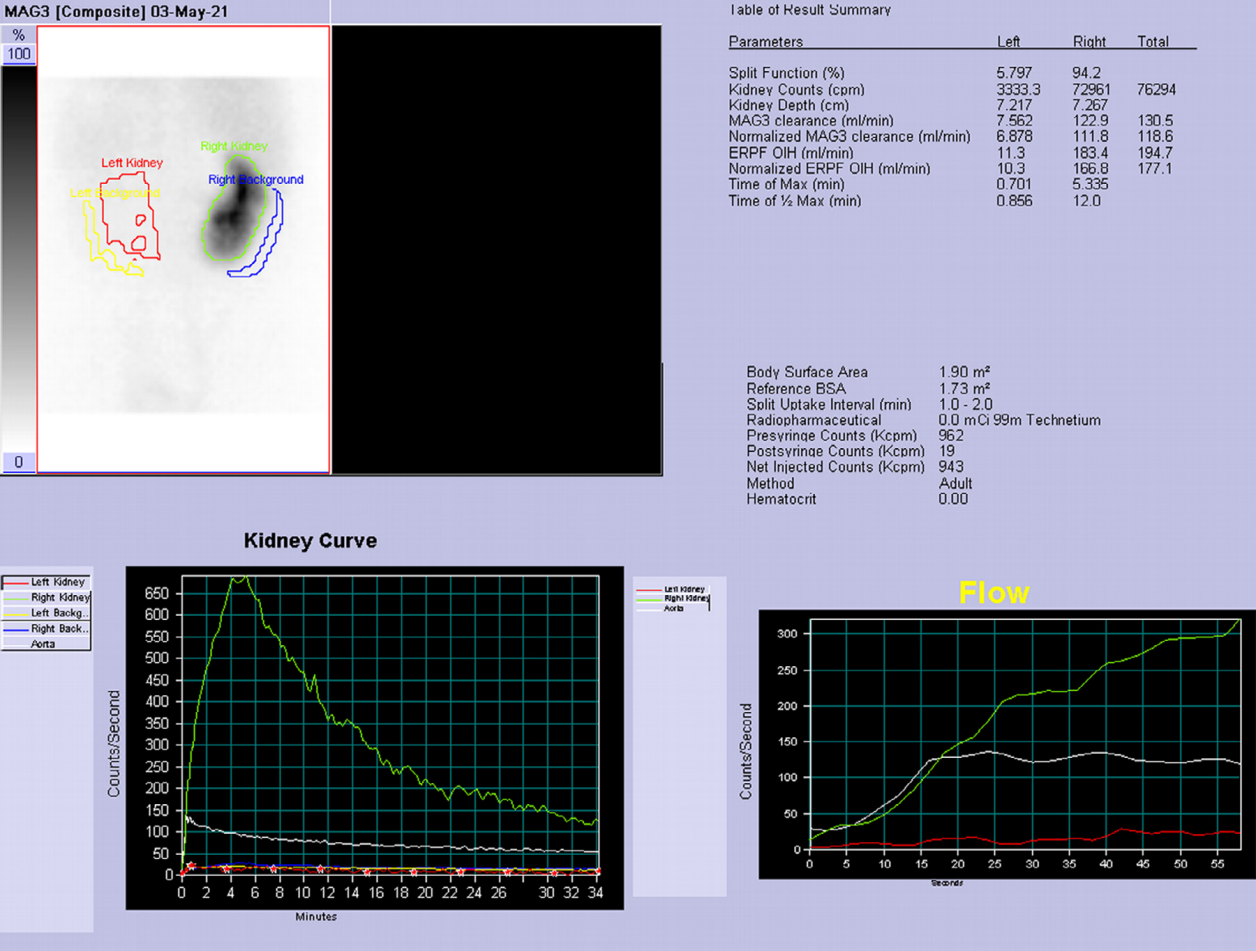
Renal scintigraphy. Nonfunctional left kidney.

A 50 years-old woman referred at our hospital complaining dry cough, abdominal pain and vomit for the past 7 days. She was afebrile and referred history of asymptomatic left kidney lithiasis. Physical examination revealed an emaciated individual, during the previous year she had lost 35 pounds and had numerous episodes of back pain, nausea, and night sweats and and repeated urinary tract infections. Pulse rate was 70 per minute; blood pressure 90/65 mm Hg, and respiratory rate 12 per minute. Dullness to percussion and decrease respiratory sounds were noted posteriorly over the left lower chest and tenderness to percussion in the left costovertebral region. Laboratory data included hemoglobin 10 g/dl, hematocrit 30.6%, white blood cell count 15,7 103/mm3, neutrophils 86,8% (normal value 40-75), creatinine 1,02 mg/dl, C reactive protein 25,81 mg/dl, procalcitonin 0.17 (low risk >0.5) Urinalysis revealed urinary leukocyte esterase activity 500 (Leu/ul), trace of hemoglobin 0.10 mg/dl, proteins 70 mg/dl and many gram negative rods. A chest x-ray film showed a small area of infiltration on the posterobasal zone of the left hemidiaphragm (Figs. 1A and B). Abdominal ultrasound detected an enlarged left kidney with dilated calico-pelvic system fluid filled by inhomogeneous hypoechoic material and a staghorn calculous. The fluid filled calico-pelvic system appeared continuing into a loculated collection extending above the perirenal fascia with associated inhomogeneity of perirenal fat (Fig. 2A-D). A suspected ultrasound diagnosis of complicated pyonephrosis was formulated, and Computed Tomography (CT) of chest, abdomen and pelvis with intravenous contrast was performed in order to stage and define the extension of the pathology [3]. CT with intravenous contrast confirmed the presence on and enlarged kidney with staghorn calculous and markedly dilated fluid filled calico-pelvic system . The calico-pelvic dilated system continued into a fluid collection in the perirenal fat at the upper pole of the kidney, with hyperemic wall referable to a perinephic abscess (Fig. 3A-C). The perinephric abscess was connected to bronchi through a fistulous tract that passed thorough the left hemidiaphragm (Fig. 4A). Bronchi involved into the fistulous tract appeared enlarged with thickened and irregular walls, they appeared air filled, and no endobronchial fluid stasis was detected (Fig. 5A-C). A diagnosis of complicated pyonephrosis with perinephric abscess and nephrobronchial fistula was formulated. The patient was admitted to the hospital and treated with antibiotic therapy (merren intravenous 1 gr x 3, bactrim 800/160 1 vial x 2). A left tube nephrostomy was placed. Six days after the placement of nephrostomy and intravenous antibiotic therapy, laboratory data (white blood cells 7,6 103/mm3, neutrophils 69,2 %, C reactive protein 2,99 mg/dl, procalcitonin 0.04 ng/dl-low risk <0.5) showed a significantly improvement of the inflammatory status. Follow up CT was performed after 15 days from the admission, CT findings demonstrating considerable reduction of the dilatation of calico pelvic system and of the perinephric abscess at the upper pole (Fig. 6A-C). Moreover the fistulous tract was not appreciable anymore, instead an inhomogeneous tissue with moderate contrastographic enhancement was detected, it was referred to the possibly presence of reparative tissue, and the healing of the fistulous connection was suspected at CT (Figs. 4A and B). Renal scintigraphy was performed, demonstrating a nonfunctioning left kidney (Fig. 7). Left nephrectomy was performed under general anesthesia. At surgery, a high lumbar incision was made with partial sub- periostic extraction of the 12th rib. Extra-gerotal dissection was done all around the kidney, there were dense perinephric adhesion especially toward the upper pole, tracking toward the diaphragm. Careful dissection of all adhesions was done up. On separation of the upper renal pole there was an escape of pus and a fistulous communication was found between the upper renal pole and the left diaphragm, the fistulous wound was appreciable and appeared healed, confirming follow up CT findings. The fistulous tract was excised flus with the diaphragm, the diaphragmatic ren was closed with reinforcement by a pad of fat. Subdiaphragmatic drain was placed. The healing of the fistula avoided any pulmonary complications during surgery [10,^12^,^15^,^25^,26]. Histological examination of the kidney showed xanthogranulomatous pyelonephritis. Postoperative course was unremarkable.

## Discussion

Complicated infectious renal diseases can determine the spread of an intrarenal suppurative process into perirenal space with involvement of the Gerota's fascia; perinephric abscess caused by blood-born infection [19] or extension of disease of adjacent organs are less common. The most affected structure includes duodedum, colon, diaphragm, and thoracic cavity overlying each diaphragm. An anatomic explanation as to how a perinephric suppurative process extends up through the diaphragm was given by Evans [5], who hypothesize tha the anterior and the posterior perirenal fascial layers meet and attach to the diaphragm fascia above the adrenal glands [14,27] and the point of perirenal fascia attachment to the diaphragm is usually the point of eventual perforation and communication with the pleural cavity and the lung [5,13].

Pyonephrosis and xanthogranulomatous pyelonephritis confined to the perinephric area by perirenal fascia may be indolent and remain occult, symptoms may appear in the advanced stages when the suppurative lesion disrupt the surrounding perirenal fascia and involve the adjacent anatomic structure [3,^4^,[28], [29], [30]. Early recognition of patients with a perinephric abscess may be difficult and symptoms often are non-specific [10].

Xanthogranulomatous pyelonephritis (XP) is a rare and severe chronic infection of renal parenchyma seldom encountered in clinical practice [19]. It is usually characterized by renal obstruction in which repeated renal infection and inflammation not revealed and treated, evolve toward diffuse renal destruction. XP can present as a diffuse form (90%) or focal/tumefactive form (10%) [31,32]. When there is renal parenchyma necrosis and destruction, formation of fistulas between kidney and adjacent organs or structures occur [19,^29^,^31^,33]. For its local and regional spread and extension, xanthogranulomatous pyelonephritis is staged based on the degree of involvement of the adjacent tissue: stage I, the disease is confined to the renal parenchyma only; stage II, the disease involves renal parenchyma as well the perirenal fat; stage III, the disease extend into the perirenal and pararenal spaces or diffuse retroperitoneum [34]. The most affected structure includes duodenum, colon, diaphragm, and thoracic cavity overlying each diaphragm. Nephrobronchial fistula were rare event before the antibiotic era. Nowadays urinary tract infections still represent a severe public health problem and patients suffering from symptomatic UTI are commonly treated with antibiotics, these treatments can result in long-term alteration of the normal micro-biota and determine the development of multidrug-resistant microorganism, in this scenarios clinician should be prepared to consider such rare complications [35,36]. Our literature review performed using PubMed database revealed 30 published case reports for a total of 31 cases of nephrobronchial fistula reported after 1949; 21 full papers were available and reviewed for a total of 21 patients with nephrobronchial fistula (17 female/4 male), age range 12-68 years-old (median 45,68 y/o). The side affected was the left in 14 patients and the right side in 7 patients. Cause reported were infections (3/21) [6,^7^,23], pyonephrosis (8/21) [9], [10], [11], [12], [13], [14], [15],25], xanthogranulomatous pyelonephritis (10/21) [16,^17^,^26^,37]. Pathogens reported in infections and pyonephrosis were *Escherichia Coli* (2/11), *Proteus Mirabilis* (6/11), tuberculosis (1/11), *Pseudomonas Aeruginosa* (1/11), *Staphylococcus Pyogenes* (1/21). In 2 patients *Proteus Mirabilis* was associated with *Pseudomonas Aerug*inosa and in another patient with *Providencia Rettgeri*.

The symptoms of perinephric abscess include fever and chills, unilateral flank pain or tenderness in the back, generalized abdominal pain, night sweats, weight loss. Patients with this condition may have symptoms referable to the urinary tract, although pulmonary symptoms may dominate causing the urinary tract disease overlooked completely. Pulmonary symptoms usually referred are chest pain, cough, copious foul-smelling sputum and productive cough, and sometimes patients suffered of uroptosis, a urine-like taste in the mouth. Based on our literature research, 10 of 21 patients presented respiratory symptoms: 3 of 10 with cough [7,^10^,16], 5 of 10 with productive cough and foul smelling sputum [12,[18], [19], [20], [21], 1 of 10 with chest pain [14], and 1 patients with hemoptysis [22]. Only 1 patient presented with productive cough and associate urinary symptoms [13]. Nine of 21 patients referred urinary symptoms [6,^9^,^11^,^15^,^17^,^23^,^25^,^37^,38]. One patients had a pus discharging sinus in the left flank [26]. The admission history and physical examination are helpful when they reveal the classic symptoms of a primary genitourinary or cutaneous infection, followed by the onset of fever and flank pain, unfortunately, such a history is obtainable in only about 50% of patients. This may explained by the fact that the renal infection may be confined to the perinephric area by perirenal fascia, and it may be indolent and remain occult, becoming symptomatic only when the suppurative lesion disrupts the surrounding perirenal fascia and the process extends to involve the adjacent anatomic structure. Nephrobronchial fistula is an extremely rare event, patients often present with respiratory symptom, cough and productive cough are the more frequent symptoms, whereas the renal infectious process may be silent in half of patients. Awareness of this entity and its presentation should help to prevent misdiagnosis or delayed diagnosis when this unusual condition is encountered. The surgical treatment of a nephrobronchial fistula is nephrectomy and appropriate drainage of the perinephric abscess, with the adequate drainage the fistula tract does not need to be operate and additional surgery is not needed [14,17]. These patients typically respond well to drainage and antibiotic therapy but often require nephrectomy, as in our case. Based on our previously reported experience [4], the presence of ipsilateral chest lesion in patient with renal disease should alert the clinician of the possibility of a pulmonary extension of the renal infectious process. Our case confirmed data previously published, nephrobronchial fistula was found in left kidney stage III xanthogranulomatous pyelonephritis in a middle age immunocompetent woman who presented with respiratory symptom and underlying renal disease. The efficacy of antibiotic therapy determined the healing of the fistulous connection, and the patient underwent nephrectomy with no respiratory complication during intubation.

## Conclusion

The most common cause in the formation of a nephrobronchial fistula is a preexisting perinephric abscess, in patients with renal infectious disease an ipsilateral postero-basal pulmonary infiltrate should alert the clinician to consider renal abnormality as a cause o of lung complication even in the absence of urinary symptoms.

## Patient consent

Patient's consent not required as patient's identity is not disclosed or compromised.
